# Influence of the endocannabinoid system on the antidepressant activity of bupropion and moclobemide in the behavioural tests in mice

**DOI:** 10.1007/s43440-020-00088-0

**Published:** 2020-03-27

**Authors:** Ewa Poleszak, Sylwia Wośko, Karolina Sławińska, Elżbieta Wyska, Aleksandra Szopa, Katarzyna Świąder, Andrzej Wróbel, Jarosław Szponar, Urszula Doboszewska, Piotr Wlaź, Aleksandra Wlaź, Anna Serefko

**Affiliations:** 1 Laboratory of Preclinical Testing, Chair and Department of Applied and Social Pharmacy, Medical University of Lublin, Chodźki 1, 20-093 Lublin, Poland; 2 Chair and Department of Applied and Social Pharmacy, Medical University of Lublin, Chodźki 1, 20-093 Lublin, Poland; 3 Department of Pharmacokinetics and Physical Pharmacy, Jagiellonian University Medical College, Medyczna 9, 30-688 Kraków, Poland; 4 Second Department of Gynecology, Medical University of Lublin, Jaczewskiego 8, 20-090 Lublin, Poland; 5 Toxicology Clinic, Medical University of Lublin: Clinical Department of Toxicology and Cardiology, Stefan Wyszyński Regional Specialist Hospital in Lublin, Al. Kraśnicka 100, Lublin, Poland; 6 Department of Animal Physiology, Faculty of Biology and Biotechnology, Maria Curie-Skłodowska University, Akademicka 19, 20-033 Lublin, Poland; 7 Department of Pathophysiology, Medical University of Lublin, Jaczewskiego 8, 20-090 Lublin, Poland

**Keywords:** Oleamide, AM251, JWH133, AM630, Forced swim test, Tail suspension test

## Abstract

**Background:**

Though there are several classes of antidepressant drugs available on the pharmaceutical market, depression that affects globally over 320 million people is still undertreated. Scientists have made attempts to develop novel therapeutical strategies to maximize effectiveness of therapy and minimize undesired reactions. One of the ideas is use of either dual-action agents or combined administration of two substances that affect diverse neurotransmissions. Thus, we investigated whether the selected CB receptor ligands (oleamide, AM251, JWH133, and AM630) can have an impact on the activity of bupropion and moclobemide. Bupropion belongs to the dual acting drugs, whereas moclobemide is an inhibitor of monoamine oxidase.

**Methods:**

The mice forced swim test and the tail suspension test were applied in order to determine the potential antidepressant-like activity, whereas the HPLC method was used in order to assess the brain concentrations of the tested antidepressants.

**Results:**

An intraperitoneal injection of sub-effective doses of oleamide (5 mg/kg), AM251 (0.25 mg/kg), and AM630 (0.25 mg/kg) increased activity of bupropion (10 mg/kg) in both behavioural tests. Effects of moclobemide (1.5 mg/kg) were potentiated only by AM251. These results were not influenced by the hypo- or hyperlocomotion of animals.

**Conclusion:**

The outcomes of the present study revealed that particularly activation or inhibition of the CB_1_ receptor function may augment the antidepressant activity of bupropion, whereas only inhibition of the CB_1_ receptor function manages to increase activity of moclobemide. Most probably, an interplay between CB receptor ligands and bupropion or moclobemide takes place at the cellular level.

## Introduction

Though there are several classes of antidepressant drugs available on the pharmaceutical market, depression that affects globally over 320 million people is still undertreated. Not only insufficient efficacy of drugs is responsible for such a situation, but also a high rate of remissions, side effects, and patient’s non-compliance. Epidemiological data have estimated that even up to 40% and up to 60% of depressed patients do not respond to the introduced antidepressant therapy and do not achieve complete remission, respectively [1]. Moreover, up to 50–85% of them experience relapses of the disease. Another great disadvantage of the approved antidepressants is their delayed onset of action—usually one should wait at least 2 weeks since the beginning of therapy to observe an improvement of clinical symptoms. Delayed onset of pharmacological action plus occurrence of adverse effects may encourage a depressed patient to abandon their treatment [2]. Therefore, over the last few decades scientists have made attempts to develop novel antidepressant drugs and/or therapeutical strategies to maximize effectiveness of therapy and minimize undesired reactions. Since the pathophysiology of depression is multifactoral, one of the ideas is use of either dual-action agents or combined administration of two substances that affect diverse neurotransmissions (i.e. serotoninergic, dopaminergic noradrenergic, melatoninergic signalling) [3, 4]. Moreover, current experimental pre-clinical and clinical treatments for depression focus on substances with novel mechanisms of action targeting different pathways implicated in the pathogenesis of depression, including the stress axis, neurogenesis, inflammatory processes, oxidative stress, and glutamatergic, opioid, cholinergic, or endocannabinoid neurotransmissions. New compounds are tested both as monotherapy and as enhancers of the conventional antidepressant drugs [5]. When designing the present experiments, we decided to concentrate on the second option. Knowing that cannabinoid (CB) receptor ligands are able to potentiate the activity of common antidepressants, i.e. the tricyclic imipramine, selective serotonine reuptake inhibitor—escitalopram, and selective inhibitor of noradrenaline reuptake—reboxetine [6], we wanted to check whether they can also affect the effects of other antidepressant drugs that have a little bit different biological targets, such as bupropion and moclobemide. Bupropion belongs to the dual acting antidepressant agents, and it acts via inhibition of reuptake of dopamine and noradrenaline. Moclobemide is a representative of the reversible and selective inhibitors of monoamine oxidase. As CB receptor ligands, we selected substances with documented antidepressant-like potential, i.e. oleamide and AM251—an agonist and inverse agonist/antagonist of CB_1_ receptors, as well as JWH133 and AM630—an agonist and inverse agonist/antagonist of CB_2_ receptors, respectively. Though moclobemide seems to produce rapid and significant improvement in the quality of life of people with depression, it should not be used concomitantly with other drugs that enhance the serotoninergic neurotransmission due to the risk of the serotonergic overactivity (i.e. hyperthermia, confusion, hyperreflexia and myoclonus) [7]. On the other hand, bupropion, which is frequently prescribed as a component of a combined antidepressant therapy, may cause adverse reactions related to dopamine over-stimulation (including nausea, insomnia, agitation, dry mouth, weight loss, psychosis, and the lowered seizure threshold) [8]. We assume that the combination of moclobemide or bupropion with substances modifying the endocannabinoid system may improve the clinical effect of these agents and/or improve their safety profile (as a result of dose reduction) [9]. In order to investigate the influence of CB receptor ligands on the activity of bupropion and moclobemide, we performed two worldly recognized behavioural tests that are widely used for evaluation of the antidepressant potential, i.e. the mouse forced swim test (FST) and the mouse tail suspension test (TST). Additionally, we performed the pharmacokinetic analysis to determine whether the CB receptor ligands affect concentrations of the tested antidepressants in the mouse brain.

## Materials and methods

### Animals

The presented experiments were carried out on adult male Albino Swiss mice weighing about 25–30 g. Animals were housed in standard cages (8 mice/cage) with free access to food and water. Rooms at the animal facility were environmentally controlled, with temperature of 22–23 °C, relative humidity of 45–55%, and with 12-h light/dark cycle. 7–10 subjects represented one experimental group. The research was planned and executed in agreement with Polish and European law related to studies on laboratory animals, and the applied procedures were approved by the Local Ethics Committee.

### Drugs

All CB receptor ligands, i.e. oleamide (*cis*-9,10-octadecenoamide, 5 mg/kg, Tocris), AM251 (*N*-(piperidin-1-yl)-5-(4-iodophenyl)-1-(2,4-dichlorophenyl)-4-methyl-1*H*-pyrazole-3-carboxamide, 0.25 mg/kg, Tocris), JWH133 ((6aR,10aR)-3-(1,1-dimethylbutyl)-6a,7,10,10a-tetrahydro-6,6,9-trimethyl-6H-dibenzo[b,d]pyran, 0.25 mg/kg, Tocris), and AM630 (6-iodo-2-methyl-1-[2-(4-morpholinyl)ethyl]-1*H*-indol-3-yl](4-methoxyphenyl)methanone, 0.25 mg/kg, Tocris), were suspended in Tween 80 solution (1%). Bupropion (10 mg/kg, Abcam) and moclobemide (1.5 mg/kg, Sigma-Aldrich) were dissolved in saline. Both CB receptor ligands and antidepressant drugs were given intraperitoneally (*ip*) 30 and 60 min before behavioural testing, respectively. Mice from the control groups were injected with either 1% aqueous solution of Tween and/or saline. The pre-treatment schedules and the tested doses were chosen on the basis of the literature data [10] and our previous experimental projects [6, 11, 12]. A standard volume of liquid dosage forms (i.e. 10 ml/kg) was used.

### Forced swim test (FST)

The FST was carried out according to the same protocol which we had applied previously [13]. Duration of immobility, i.e. the time when mice stopped struggling and remained floating, performing only movements necessary to keep its head above the water level, was measured for the last 4 min of the 6-min experiment.

### Tail suspension test (TST)

The TST was carried out according to the same protocol which we had applied previously [13]. Duration of immobility, i.e. the time when mice stopped struggling, performing only movements necessary to breathe, was measured for the last 4 min of the 6-min experiment.

### Spontaneous locomotor activity

The spontaneous locomotor activity was recorded automatically with use of the animal activity meter Opto-Varimex-4 Auto-Track (Columbus Instruments, USA), according to the same protocol which we had applied previously [13]. A travelled distance was measured for the last 4 min of the 6-min experiment, which corresponded with the testing period taken into account the FST and the TST.

### Pharmacokinetic assays

The murine brain levels of bupropion and moclobemide were determined according to the same protocol which we had applied previously by a high-performance liquid chromatography (HPLC) method [11]. Animals were decapitated 60 min after injection of bupropion or moclobemide (given with or without the respective ligand of CB receptors). The tests were reproducible with low intra- and interday variation. (Coefficient of variation was less than 10%.) The extraction efficiencies of the analysed drugs and the internal standard ranged from 66 to 97%. Concentrations of bupropion and moclobemide were given for wet brain tissue in ng/g.

### Statistical analysis

Statistical analysis of the obtained results was performed by either two-way analysis of variance (ANOVA) with Bonferroni’s multiple comparisons test or *t* test. Two-way ANOVA was used for the FST, TST, and measurements of the spontaneous locomotor activity, whereas *t* test was used for the pharmacokinetic studies. The outcomes were given as the mean ± standard error of the mean (SEM). When *p* was lower than 0.05, between-group differences were considered as significant.

## Results

### Effects of a combined administration of the CB_1_ receptor ligands and bupropion in the FST and the TST

**Fig. 1:**
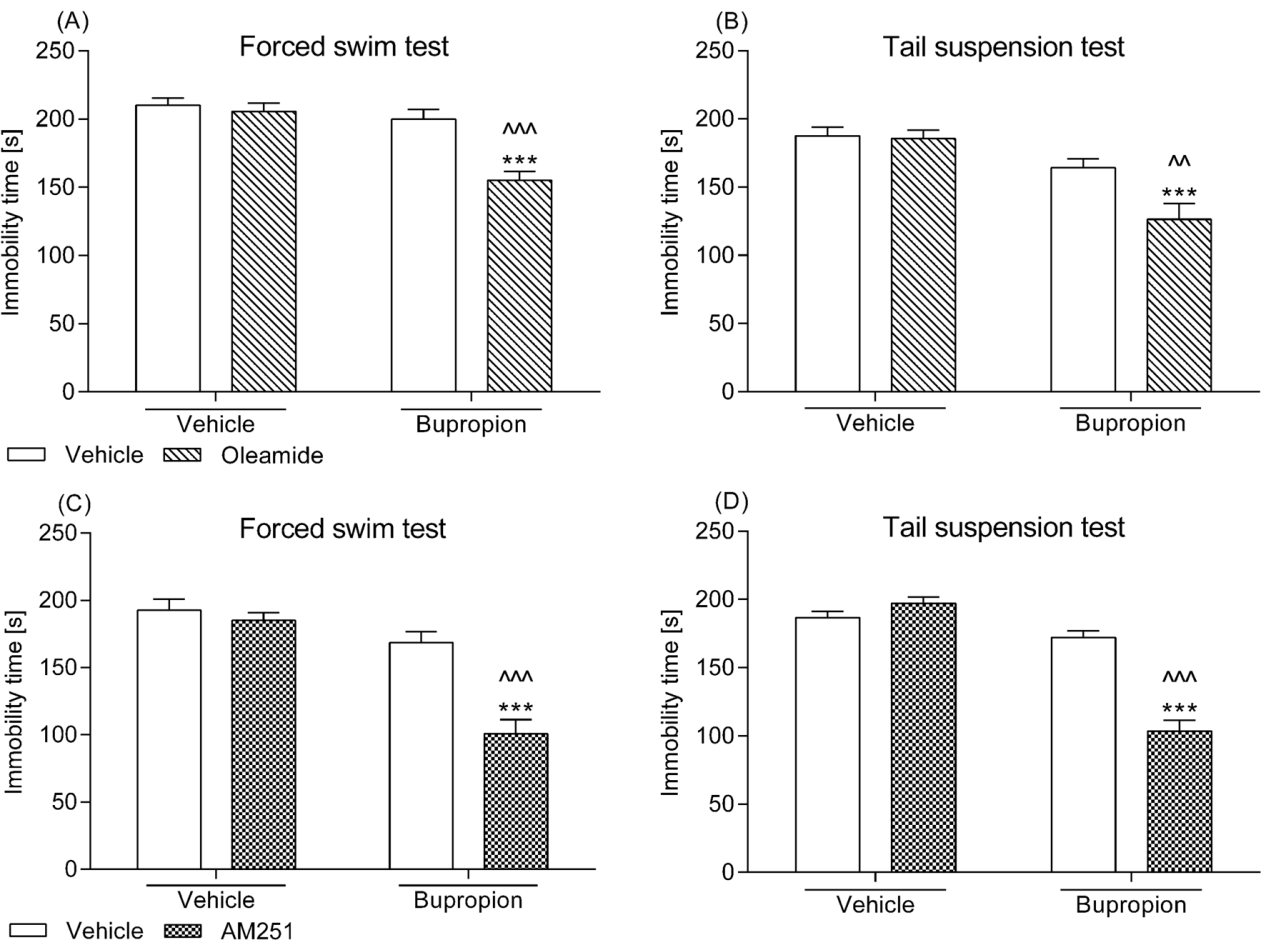
Effect of a combined intraperitoneal administration of oleamide or AM251 and bupropion in **a**, **c** the FST and **b**, **d** the TST in mice. Oleamide (5 mg/kg) and AM251 (0.25 mg/kg) were injected 30 min before behavioural tests, while bupropion (10 mg/kg) was given 60 min before testing. The values represent mean + SEM (*n* = 7–10 animals per group). Two-way ANOVA was used for statistical analysis. The Bonferroni’s post hoc test showed: ***p < 0.001 versus oleamide or AM251; ^^*p *< 0.01; ^^^*p *< 0.001 versus bupropion

Neither oleamide (5 mg/kg) nor AM251 (0.25 mg/kg) or bupropion (10 mg/kg) when administered alone significantly changed behaviour of mice in the FST or in the TST. By contrast, after exposure to respective combinations, i.e. oleamide–bupropion or AM251–bupropion, the tested animals were swimming for a longer period than their control counterparts and they struggled for a longer time in the TST (Fig. 1). Accordingly, two-way ANOVA demonstrated the following statistical data for the FST: (1) a significant oleamide–bupropion interaction [*F*(1,36) = 10.22; *p *= 0.0029] with a significant effect of oleamide [*F*(1,36) = 15.29; *p *= 0.0004] and a significant effect of bupropion [*F*(1,36) = 23.33; *p *< 0.0001], (2) a significant AM251–bupropion interaction [*F*(1,27) = 12.89; *p* = 0.0013] with a significant effect of AM251 [*F*(1,27) = 20.57; *p *= 0.0001] and a significant effect of bupropion [*F*(1,27) = 42.35; *p *< 0.0001], and for the TST: (1) a significant oleamide–bupropion interaction [*F*(1,36) = 4.73; *p *= 0.0366] with a significant effect of oleamide [*F*(1,36) = 5.91; *p *= 0.0205] and a significant effect of bupropion [*F*(1,36) = 25.11; *p *< 0.0001], (2) a significant AM251–bupropion interaction [*F*(1,28) = 46.42; *p *< 0.0001] with a significant effect of AM251 [*F*(1,28) = 24.71; *p *< 0.0001] and a significant effect of bupropion [*F*(1,28) = 86.07; *p *< 0.0001].

### Effects of a combined administration of the CB_2_ receptor ligands and bupropion in the FST and the TST

**Fig. 2:**
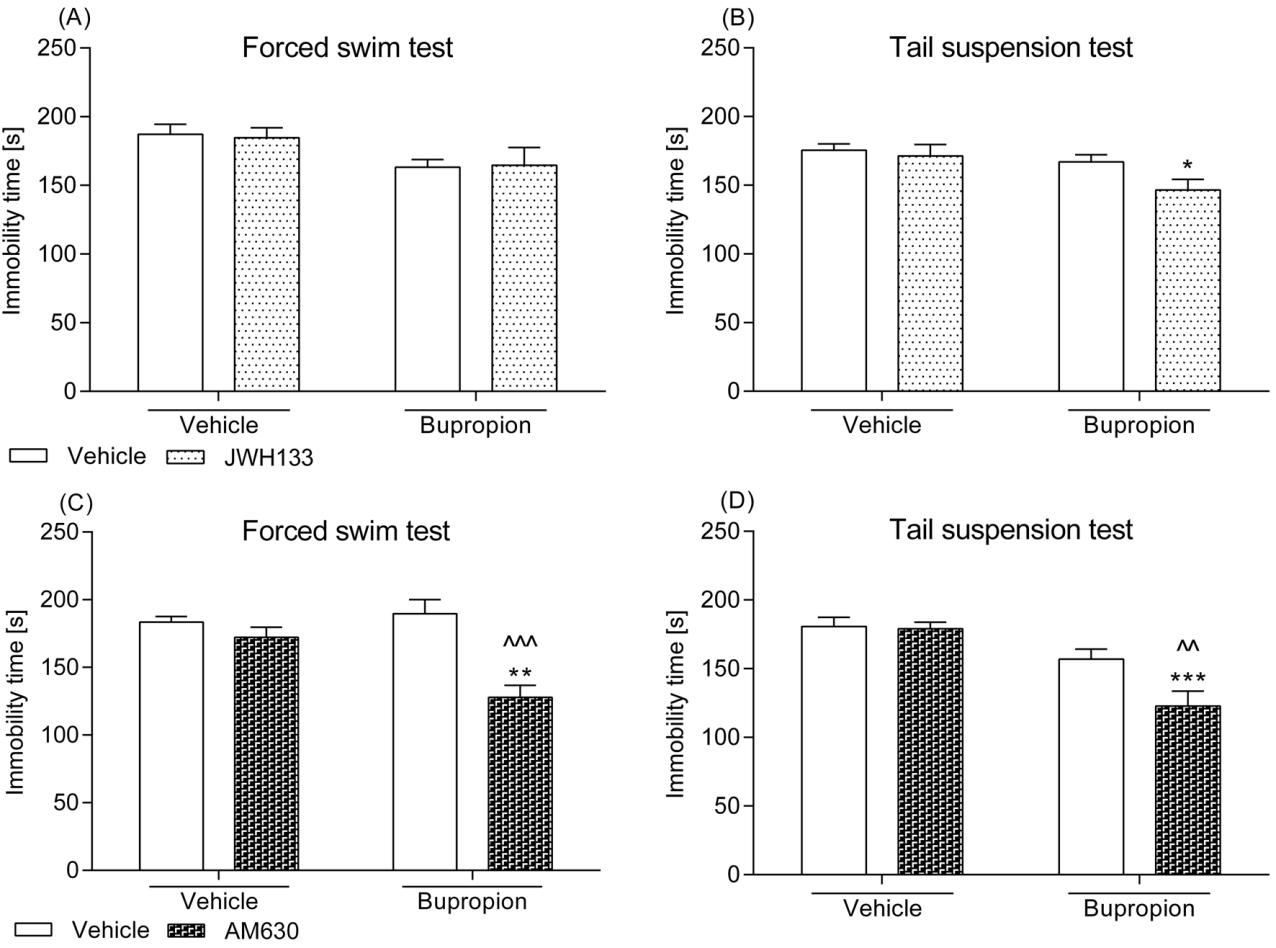
Effect of a combined intraperitoneal administration of JWH133 or AM630 and bupropion in **a**, **c** the FST and **b**, **d** the TST in mice. JWH133 (0.25 mg/kg) and AM630 (0.25 mg/kg) were injected 30 min before behavioural tests, while bupropion (10 mg/kg) was given 60 min before testing. The values represent mean + SEM (*n* = 7–8 animals per group). Two-way ANOVA was used for statistical analysis. The Bonferroni’s post hoc test showed: *p < 0.05; **p < 0.01; ***p < 0.001, versus JWH133 or AM630; ^^*p *< 0.01; ^^^*p *< 0.001 versus bupropion

As illustrated in Fig. 2, a single injection of JWH133 (0.25 mg/kg), AM630 (0.25 mg/kg), or bupropion (10 mg/kg) did not influence the swimming pattern of mice subjected to the FST. Similarly, such a treatment did not modify animals’ behaviour in the TST. Whereas concurrent administration of AM630 and bupropion produced an anti-immobility effect in both tests, the combined treatment with JWH133 and bupropion generally was not more potent than monotherapy. Statistical analysis revealed a significant drug–drug interaction for AM630 and bupropion (1) in the FST: *F*(1,28) = 9.64; *p *= 0.0043, with a significant effect of both AM630 [*F*(1,28) = 20.11; *p *= 0.0001] and bupropion [*F*(1,28) = 5.54; *p *= 0.0259], and (2) in the TST: *F*(1,28) = 4.37; *p *= 0.0457, with a significant effect of both AM630 [*F*(1,28) = 5.37; *p *= 0.0280] and bupropion [*F*(1,28) = 26.91; *p *< 0.0001]. JWH133–bupropion interaction turned out to be non-significant in the FST: *F*(1,28) = 0.06; *p *= 0.8160 and in the TST: *F*(1,27) = 1.53; *p *= 0.2262.

### Effects of a combined administration of the CB_1_ receptor ligands and moclobemide in the FST and the TST

**Fig. 3:**
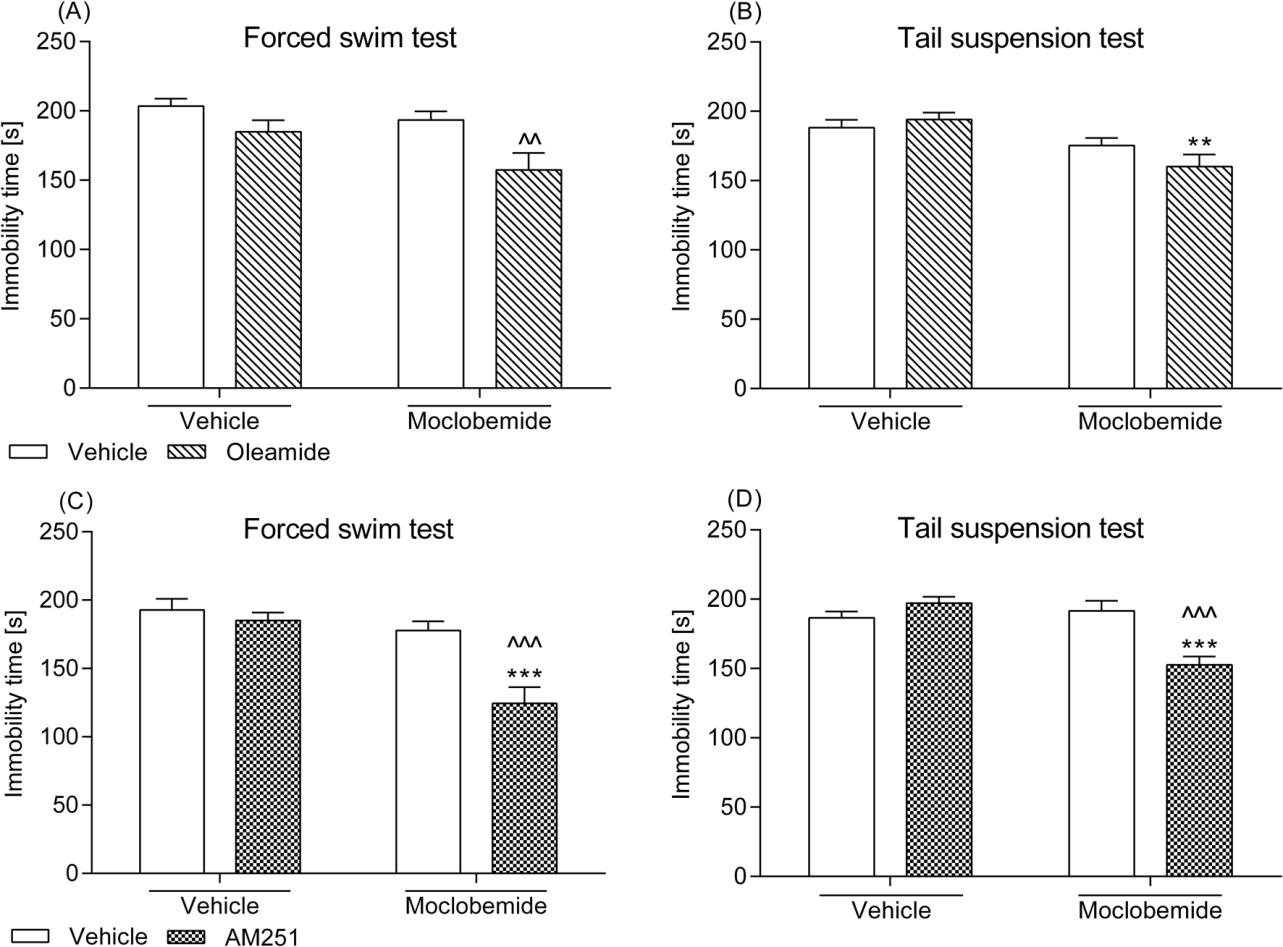
Effect of a combined intraperitoneal administration of oleamide or AM251 and moclobemide in **a**, **c** the FST and **b**, **d** the TST in mice. Oleamide (5 mg/kg) and AM251 (0.25 mg/kg) were injected 30 min before behavioural tests, while moclobemide (1.5 mg/kg) was given 60 min before testing. The values represent mean + SEM (*n* = 8–10 animals per group). Two-way ANOVA was used for statistical analysis. The Bonferroni’s post hoc test showed: **p < 0.01; ***p < 0.001 versus oleamide or AM251; ^^*p *< 0.01; ^^^*p *< 0.001 versus moclobemide

Mice given only acute single doses of oleamide (5 mg/kg), AM251 (0.25 mg/kg), or moclobemide (1.5 mg/kg) struggled for the same duration of time as the vehicle-treated ones in both applied behavioural tests. Co-administration of AM251 and the antidepressant drug prolonged the mobility time of animals in the FST and in the TST. According to calculations performed by two-way ANOVA, significant AM251–moclobemide interactions [*F*(1,28) = 8.19; *p *= 0.0122 and *F*(1,28) = 18.72; *p *= 0.0002] with significant effects of AM251 [*F*(1,28) = 13.00; *p *= 0.0012 and *F*(1,28) = 6.06; *p *= 0.0203] and significant effects of moclobemide [*F*(1,28) = 19.74; *p *= 0.0001 and *F*(1,28) = 11.82; *p *= 0.0019] were detected in the FST and the TST, respectively. Though Bonferroni’s post hoc test showed that mice receiving concurrent administration of oleamide and moclobemide struggled for a longer time than moclobemide-treated animals or oleamide-treated animals in the FST or the TST, respectively, a significant oleamide–moclobemide interaction was not detected by two-way ANOVA. The statistical data were obtained as follows: (1) *F*(1,36) = 1.07; *p *= 0.3076 for oleamide–moclobemide interplay in the FST, and (2) *F*(1,36) = 2.73; *p *= 0.1070 for oleamide–moclobemide interplay in the TST. The outcomes from the behavioural tests are presented in Fig. 3.

### Effects of a combined administration of the CB_2_ receptor ligands and moclobemide in the FST and the TST

**Fig. 4:**
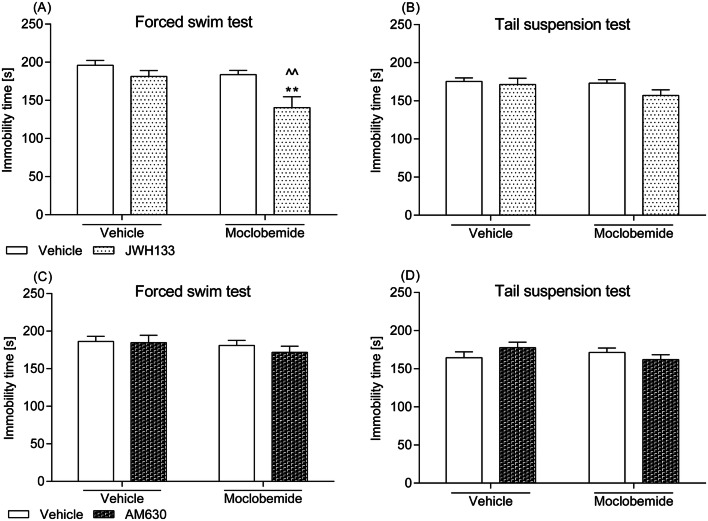
Effect of a combined intraperitoneal administration of JWH133 or AM630 and moclobemide in **a**, **c** the FST and **b**, **d** the TST in mice. JWH133 (0.25 mg/kg) and AM630 (0.25 mg/kg) were injected 30 min before behavioural tests, while moclobemide (1.5 mg/kg) was given 60 min before testing. The values represent mean + SEM (*n* = 8 animals per group). Two-way ANOVA was used for statistical analysis. The Bonferroni’s post hoc test showed: **p < 0.01 versus JWH133; ^^*p *< 0.01 versus moclobemide

The anti-immobility effect of a joint administration of per se sub-active doses of CB_2_ receptor ligands (i.e. 0.25 mg/kg of JWH133 or AM630) with moclobemide (1.5 mg/kg) was not significantly stronger than the monotherapy. Though, as shown in Fig. 4, mice given JWH133 and moclobemide struggled a little bit longer in the FST than their control counterparts, the observed differences in behaviour were not prominent enough to be detected by two-way ANOVA as an indicator of a significant drug–drug interplay. Consequently, the statistical analyses demonstrated the following results: (1) a non-significant JWH133–moclobemide interaction in the FST: *F*(1,28) = 2.45; *p *= 0.1288, (2) non-significant JWH133–moclobemide interaction in the TST: *F*(1,28) = 0.84; *p *= 0.3667, (3) non-significant AM630–moclobemide interaction in the FST: *F*(1,28) = 0.22; *p *= 0.6425, and (4) non-significant AM630–moclobemide interaction in the TST: *F*(1,28) = 2.69; *p *= 0.1125.

### Effects of a combined administration of the CB receptor ligands and bupropion or moclobemide on the spontaneous locomotor activity in mice

None of the tested agents, i.e. oleamide (5 mg/kg), AM251 (0.25 mg/kg), JWH133 (0.25 mg/kg), AM630 (0.25 mg/kg), bupropion (10 mg/kg), or moclobemide (0.15 mg/kg), given alone or in respective combinations influenced the spontaneous locomotor activity of the tested mice. (Data are presented in Table 1.) Consequently, two-way ANOVA demonstrated: (1) a non-significant oleamide–bupropion interaction: *F*(1,27) = 2.02; *p *= 0.1665, (2) non-significant oleamide–moclobemide interaction: *F*(1,28) = 0.00; *p *= 0.9607, (3) non-significant AM251–bupropion interaction: *F*(1,28) = 0.61; *p *= 0.4396, (4) non-significant AM251–moclobemide interaction: *F*(1,28) = 0.51; *p *= 0.4816; (5) non-significant JWH133–bupropion interaction: *F*(1,26) = 0.11; *p *= 0.7431, (6) non-significant JWH133–moclobemide interaction: *F*(1,28) = 2.89; *p *= 0.0999, (7) non-significant AM630–bupropion interaction: *F*(1,26) = 1.24; *p *= 0.2754, and (8) non-significant AM630–moclobemide interaction: *F*(1,28) = 1.16; *p *= 0.2904.

**Table 1:** Effect of a combined intraperitoneal administration of (A) oleamide, (B) AM251, (C) JWH133, or (D) AM630 and bupropion or moclobemide on the spontaneous locomotor activity of mice

	Treatment (*n* = number of mice per group)	Travelled distance (cm)
(A)	Vehicle + vehicle (*n* = 8)	412.1 ± 86.02
	Oleamide + vehicle (*n* = 8)	470.1 ± 64.35
	Bupropion + vehicle (*n* = 7)	683.3 ± 40.25
	Bupropion + oleamide (*n* = 8)	511.8 ± 107.27
	Vehicle + vehicle (*n* = 8)	662.6 ± 42.78
	Oleamide + vehicle (*n* = 8)	511.5 ± 83.66
	Moclobemide + vehicle (*n* = 8)	715.3 ± 55.72
	Moclobemide + oleamide (*n* = 8)	552.3 ± 212.71
(B)	Vehicle + vehicle (*n* = 8)	629.0 ± 82.22
	AM251 + vehicle (*n* = 8)	647.6 ± 47.97
	Bupropion + vehicle (*n* = 8)	833.8 ± 109.44
	Bupropion + AM251 (*n* = 8)	708.6 ± 112.13
	Moclobemide + vehicle (*n* = 8)	582.9 ± 74.32
	Moclobemide + AM251 (*n* = 8)	703.1 ± 75.61
(C)	Vehicle + vehicle (*n* = 8)	586.5 ± 49.79
	JWH133 + vehicle (*n* = 8)	567.0 ± 53.30
	Bupropion + vehicle (*n* = 8)	868.6 ± 86.04
	Bupropion + JWH133 (*n* = 8)	800.6 ± 103.6
	Moclobemide + vehicle (*n* = 8)	510.4 ± 65.53
	Moclobemide + JWH133 (*n* = 8)	670.3 ± 38.75
(D)	Vehicle + vehicle (*n* = 7)	550.3 ± 39.53
	AM630 + vehicle (*n* = 7)	572.5 ± 48.02
	Bupropion + vehicle (*n* = 8)	748.1 ± 61.70
	Bupropion + AM630 (*n* = 8)	634.5 ± 80.94
	Vehicle + vehicle (*n* = 7)	456.6 ± 72.04
	AM630 + vehicle (*n* = 8)	544.9 ± 34.57
	Moclobemide + vehicle (*n* = 8)	516.4 ± 75.66
	Moclobemide + AM630 (*n* = 8)	518.1 ± 57.95

Oleamide (5 mg/kg), AM251 (0.25 mg/kg), JWH133 (0.25 mg/kg), and AM630 (0.25 mg/kg) were given 30 min before the experiment, whereas bupropion (10 mg/kg) and moclobemide (1.5 mg/kg) were injected 60 min before testing. The values represent mean ± SEM (two-way ANOVA followed by Bonferroni’s post hoc test)

### Pharmacokinetic studies

Analysing the results of the pharmacokinetic studies, we took into consideration only these drug combinations that were effective in the behavioural tests. Thus, we found out that despite positive interaction in the FST and in the TST, neither oleamide (5 mg/kg) nor AM251 (0.25 mg/kg) or AM630 (0.25 mg/kg) influenced the brain levels of bupropion (10 mg/kg) and/or moclobemide (1.5 mg/kg). The following statistical data were obtained after applying *t* test: (1) *t*(18) = 0.5480, *p *= 0.5905 for oleamide–bupropion combination, (2) *t*(14) = 0.1109, *p *= 0.9133 for AM251–bupropion combination, (3) *t*(14) = 0.5416, *p *= 0.5966 for AM630–bupropion combination, and (4) *t*(10) = 1.842, *p *= 0.0953 for AM251–moclobemide combination. The outcomes are summarized in Table 2.

**Table 2:** Effects of CB receptor ligands on the brain levels of bupropion and/or moclobemide in mice

Treatment	Drug level in the brain (ng/g)	Number of animals per group
Bupropion + vehicle	1207 ± 130.8	10
Bupropion + oleamide	1317 ± 152.4	10
Bupropion + vehicle	1195 ± 204.0	8
Bupropion + AM251	1226 ± 189.8	8
Bupropion + vehicle	666.2 ± 88.78	8
Bupropion + AM630	597.6 ± 90.18	8
Moclobemide + vehicle	17.23 ± 1.289	5
Moclobemide + AM251	15.07 ± 0.4063	7

Oleamide (5 mg/kg), AM251 (0.25 mg/kg), and AM630 (0.25 mg/kg) were administered intraperitoneally 30 min before decapitation, whereas bupropion (10 mg/kg) and moclobemide (1.5 mg/kg) were injected intraperitoneally 60 min before decapitation. The values represent mean ± SEM (*t* test)

## Discussion

The outcomes of our study for the first time demonstrated that CB receptor ligands are able to potentiate the antidepressant activity of bupropion and moclobemide in the recognized behavioural tests in albino Swiss mice. The endocannabinoid system attracted attention of scientists as a promising target for the antidepressant therapy a long time ago. It turned out that both CB_1_ and CB_2_ receptors are localized peripherally and centrally, including these parts of the brain that are involved in emotion-related responses, such as anxiety, low mood, or stress (i.e. the hippocampus, amygdala, prefrontal cortex) [14, 15]. In fact, Smaga et al. [16] demonstrated that rats with experimentally induced childhood- or adult-like depression (i.e. the Wistar Kyoto or olfactory bulbectomized animals, respectively) presented diverse alterations in the endocannabinoid system, which included down-regulation of CB receptors, disturbances in the brain levels of anandamide and 2-arachidonoylglycerol, and changed expression of enzymes implicated in synthesis or metabolism of endocannabinoids (i.e. *N*-acyl phosphatidylethanolamine-specific phospholipase d, monoacylglycerol lipase, fatty acid amide hydrolase, diacylglycerol lipase α). Outcomes from clinical and *post*-*mortem* studies generally confirmed the pre-clinical ones, since abnormal levels of endocannabinoids were detected in serum of untreated female patients with major depression [17], whereas increased concentrations of endocannabinoids were found in the prefrontal cortex of both alcoholic [18] and depressed suicide victims [19]. Monteleone et al. [20] showed that polymorphism in the CB_1_ receptor gene may be associated with a higher susceptibility to the development of depression in humans, whereas Onaivi and colleagues [21] found out that Japanese depressed and alcoholic patients presented polymorphism in cannabinoid CB_2_ receptors. Additionally, Minocci et al. [22] demonstrated a significant association between polymorphism in the CB_2_ receptor gene and occurrence of bipolar disorders.

Despite their opposite functional activity, agonists and inverse agonists/antagonists of CB receptors, including the ones used in our study, can exert the same responses in animal behavioural tests measuring an antidepressant-like or anxiolytic-like potential. Kruk-Słomka and colleagues [10] demonstrated that both CB_2_ receptor agonist—JWH133 (0.5 and 1 mg/kg) and CB_2_ receptor inverse agonist/antagonist—AM630 (0.5 mg/kg) when given at an acute dose induced anti-immobility effects in the FST in Swiss mice. Similar activity also produced the CB_1_ receptor agonist—oleamide (10 and 20 mg/kg) [10] and inverse agonist/antagonist—AM251 administered at the single dose of (0.5 or 0.3 mg/kg) in NMRI mice [23]. Interestingly, the responses induced by oleamide and JWH133 in the FST were attenuated/reversed by per se ineffective doses of AM251 (0.25 mg/kg) and AM630 (2 mg/kg) [10].

In the present study, we showed that oleamide (5 mg/kg), AM251 (0.25 mg/kg), and AM630 (0.25 mg/kg) augmented the antidepressant activity of bupropion (10 mg/kg) in the FST and the TST, whereas JWH133 (0.25 mg/kg) did not have such a potential. Surprisingly, antidepressant effects of moclobemide (1.5 mg/kg) were considerably enhanced in both behavioural tests only by AM251 (0.25 mg/kg). Neither oleamide (0.5 mg/kg) nor JWH133 (0.25 mg/kg) or AM630 (0.25 mg/kg) increased activity of moclobemide sufficiently enough to be recognized as a significant interaction by two-way ANOVA. Since the same observations were made in two world widely used behavioural tests designed for measurement of the antidepressant-like activity of substances belonging to diverse chemical classes, our results should be considered as reliable. Moreover, we would like to emphasize that the outcomes were not confounded by hyper- or hypolocomotion of animals. The distance travelled by mice from all tested groups was comparable to the one covered by the vehicle-treated animals. Though altered motility of rodents can be expected after administration of cannabinoids [24, 25], our outcomes were in line with results published by Kruk-Słomka and colleagues [10].

It is quite interesting that the CB_1_ receptor ligands acting oppositely, i.e. oleamide and AM251, influenced the activity of bupropion in the same manner. However, we should admit that such a bidirectional potentiation of the antidepressant action by cannabinoids had been observed before in our laboratory. The results of our previous studies demonstrated that oleamide and AM251 when administered at the sub-threshold concentrations in albino Swiss mice can potentiate the antidepressant activity of imipramine (15 mg/kg), escitalopram (2 mg/kg), and reboxetine (2.5 mg/kg) [6]. They also enhanced the antidepressant-like properties of biometals (i.e. magnesium, 10 mg/kg and zinc, 5 mg/kg) inhibiting the glutamatergic neurotransmission [12]. Additionally, Kruk-Słomka et al. [10] reported that all of the CB receptor ligands used in the present experiments augmented the anti-immobility effects of anticholinergic scopolamine (0.3 mg/kg) in the murine FST. Apart from the antidepressant-like effects, endo- and exocannabinoids are also able to produce the depressogenic (as well as anxiogenic and anxiolytic) ones in pre-clinical studies [26]. A similar trend can be observed in cannabis users—some of them confirm beneficial effects of cannabis in the depressive disorders [27], whereas the others experience a higher number of depressive symptoms [28]. Though scientists admit that the exact mechanism of this peculiar bidirectional activity of the CB_1_ receptor ligands has not been discovered yet, they propose several explanations for this phenomena: (1) influence of testing conditions (i.e. animal strain, applied dose, environmental factors) that can induce either depressive- or antidepressant-like changes in the endocannabinoid system [29], (2) existence of the functional pools of CB receptors that are responsible for manifestation of the antidepressant or pro-depressive effects [26], (3) influence of other, non-CB_1_ and non-CB_2_, cannabinoid receptors [23], or (4) specific localization of the CB_1_ receptors in the opposing, i.e. inhibitory (GABA-ergic) and excitatory (glutamatergic) terminals [30].

We assume that the observed oleamide–, AM251–, and AM630–bupropion interactions as well as the AM251–moclobemide interaction may be the result of enhanced monoaminergic neurotransmission in the brain. CB_1_ receptors influence release of acetylcholine, dopamine, glutamate, γ-aminobutyric acid, noradrenaline, serotonin, and different hormones that are involved in the depression-related behaviour. CB_2_ receptors reduce secretion of pro-inflammatory and enhance secretion of anti-inflammatory cytokines, whereas the immune responses are also implicated in the pathogenesis of depression [24, 31, 32]. Additionally, cannabinoids are known to have an impact on the activity of adreno- and serotoninergic receptors [32–34] as well as on the functioning of SERT and NAT, i.e. the serotonin and noradrenaline transporters, respectively [32, 35]. When given at high doses, they can inhibit the activity of monoamine oxidase [36]. We guess that the interplay between the cannabinoids applied in the present study and bupropion could have been particularly attributed to the intensified dopamine-related mechanisms. Bupropion is a dual inhibitor of dopamine and norepinephrine reuptake, and stimulation of CB receptors affects the secretion of dopamine [37]. However, it should be noted that the observed results are not a consequence of the simple potentiation of dopamine activity in the brain. As described in the medical literature [38], CB_1_ receptor ligands can either increase or decrease dopamine synthesis and dopamine release. Thus, the final effect of enhanced dopamine neurotransmission is most probably a result of a complex interplay between the endocannabinoid system and other neurotransmitters (such as GABA, glutamate, and others). It is difficult to say whether the AM630–bupropion interaction was also at least partially attributed to the CB_1_ receptor-dependent mechanisms or not. One the one side, several authors noted that AM630 can behave not only as a CB_2_ receptor inverse agonist/antagonist, but it can also act as a CB_1_ receptor inverse agonist [39]; thus, it may exert similar effects to AM251. On the other hand, JWH133, which does not bind exclusively to CB_2_ receptors but also has a low affinity to CB_1_ receptors [40], did not affect the activity of bupropion. Actually, since AM630 and JWH133 act oppositely at CB_2_ receptors, their different activity towards antidepressant effects of bupropion could have been expected. Furthermore, a significant functional selectivity of the CB_2_ receptor ligands found by several independent research teams [41–43] could have contributed to the observed interplays in the behavioural studies. It was revealed that certain CB_2_ receptor ligands may activate diverse downstream intracellular pathways or may activate the overlapping ones but with different potency.

As for the AM251–moclobemide synergism of action, the potentiation of the serotoninergic signalling may be responsible. Monoamine oxidase inhibitors inhibit metabolism of serotonin and sympathomimetic amines, whereas AM251 does not directly interact with adrenergic receptors but it interplays with the serotoninergic ones. According to the literature data [44], the 5HT_1A_ receptors seem to be involved in the behavioural effects of AM251 treatment. Furthermore, Hill et al. [45] demonstrated that inhibition of monoamine oxidase (by tranycypromine) influenced cannabinoid receptor binding and changed the levels of endocannabinoids. We think that other mechanisms can also contribute to the drug–drug interactions observed in our study, since AM251 modulates the opioid signalling [23], whereas oleamide interplays with serotoninergic 5-HT_2C_ and 5-HT_7_ receptors and affects the benzodiazepine receptor- and vanilloid receptor-dependent transmissions [46]. All of these pathways are directly and/or indirectly involved in the mood control.

Pharmacokinetic analyses carried out in the present study allowed us to assess concentrations of bupropion and moclobemide in mice brains after their combined administration with CB receptor ligands. This approach was aimed at determining drug–drug interactions involving changes in drug disposition. Augmentation of bupropion and/or moclobemide levels in the brain might have been an indication of the facilitated transport of these drugs through the blood–brain barrier [47] in the presence of alterations in the endocannabinoid signalling. However, though oleamide, AM251, and AM630 intensified the anti-immobility responses recorded after administration of bupropion, none of them augmented the brain levels of this antidepressant drug. Similarly, potentiation of moclobemide effects in the FST and in the TST by AM630 was not accompanied by an increase of moclobemide concentration in the mice brain. Based on the above-mentioned results we believe that the drug–drug interactions detected in the behavioural tests were due to processes at the cellular level, so they are pharmacodynamic in nature. Molecular studies are needed to explain the observed synergism of action.

The positive interaction between CB receptor ligands and bupropion and/or moclobemide found out in our study seems to be important from the clinical point of view, since concurrent administration of antidepressants and agents affecting the endocannabinoid system (particularly via CB_1_ receptors) may improve the safety profile of the introduced treatment (due to dose reduction of either substances). Based on the literature data [9], we can also hypothesize that introduction of such a drug–drug combination may accelerate the alleviation of depressive symptoms (due to the fast onset of cannabinoids biological activity). However, further tests are needed to verify this supposition.

## Conclusion

The outcomes of the present study for the first time demonstrated that both stimulation and inhibition of the CB_1_ receptor function may intensify the antidepressant effects of bupropion, whereas only inhibition of CB_1_ receptors potentiates activity of moclobemide. Though the effects of bupropion were also enhanced by administration of the selective CB_2_ receptor inverse agonist/antagonist AM630, the observed interaction could have been partially attributed to the CB_1_ receptor-dependent mechanisms. The present study provides further evidences that addition of agents influencing the endocannabinoid system to the conventional antidepressant therapy may be a good strategy for patients resistant to currently available drugs. However, we realize that more advanced experiments are necessary to confirm the validity of such an approach.
